# Neurologists’ understanding of reproductive medicine options for genetic forms of motor neuron disease

**DOI:** 10.1080/21678421.2024.2416677

**Published:** 2024-10-18

**Authors:** Shanice Allen, Jade Howard, Christopher J Mcdermott, Felicity Boardman, Alisdair Mcneill

**Affiliations:** 1 Division of Neuroscience, Neuroscience Institute, the University of Sheffield, Sheffield, UK; 2 Academic Directorate of Neuroscience, Sheffield Teaching Hospitals NHS Foundation Trust, Sheffield, UK; 3 University of Warwick, Warwick, UK; 4 Sheffield Clinical Genetics Department, Sheffield Childrens Hospital NHS Foundation Trust, Sheffield, UK

**Keywords:** MND, preimplantation genetic testing, prenatal testing, clinicians

## Abstract

**Objectives:**

To examine the knowledge, confidence and practice of motor neuron disease (MND) clinicians toward discussing reproductive options with people who carry a causal variant in an MND gene (both clinically affected and asymptomatic).

**Methods:**

An online cross-sectional survey was distributed nationwide to UK MND clinicians and clinical geneticists and genetic counselors. The survey assessed respondents’ understanding on reproductive medicine techniques; their confidence in discussing reproductive medicine options and their access to information resources.

**Results:**

Seventy six clinicians responded to the online survey (45 neurology clinicians and 31 clinical geneticists). MND clinicians had limited knowledge and low confidence in discussing reproductive medicine options. Geneticists were more likely to carry out reproductive genetic counseling with very few MND clinicians reporting undertaking these discussions. Further, 57% of the 45 MND clinicians surveyed reported to have never made a referral for reproductive genetic counseling. Multiple barriers to offering reproductive counseling or referral were identified including a lack of knowledge, lack of awareness of the different options, lack of clinic time and uncertainty around issues such as funding for PGT and whose responsibility it comes under.

**Conclusions:**

There is a need for training and education on reproductive options and referral for these options needs to be integrated within the health system. Developing more resources for both clinicians and patients is required as MND clinicians reported a lack of resources.

## Introduction

An identifiable monogenic cause is found in around 10-20% of people with apparently sporadic motor neuron disease (MND, also known as amyotrophic lateral sclerosis (ALS)) (plwMND), and 60-80% who have a family history (1). The majority of these are autosomal dominant genes, with a 50% chance of transmission to offspring. The risk of developing MND in these individuals varies depending on the gene involved and family history (2). The most commonly identified monogenic cause in Western Europeans is the c9orf72 hexanucleotide expansion (3). In addition to the risk of MND, this expansion carries a notable association with frontotemporal dementia (4). Of therapeutic significance are missense variants in SOD1. Toferesen, an antisense oligonucleotide targeting SOD1, has demonstrated both clinical and biomarker evidence of efficacy in slowing MND progression (5). MND is genetically heterogeneous, with varying degrees of evidence for association with >20 genes (6). The large number of potentially causal genes, phenotypic heterogeneity and variable penetrance complicate genomic diagnosis and counseling.

When a causal genetic variant is identified, reproductive options such as preimplantation genetic testing (PGT) and prenatal testing (PNT) can be utilized to avoid transmission to children. PGT involves genetically testing embryos that are created via in vitro fertilization (IVF) at the blastocyst stage for the disease causing genetic variants, and only unaffected embryos are transferred into the uterus. PNT involves testing an established pregnancy to see if the fetus carries the identified causal gene variant. Chorionic villus sampling (CVS) and amniocentesis are two methods of PNT. Amniocentesis involves sampling amniotic fluid and it is usually carried out between weeks 15 and 20. CVS involves taking a tissue sample (chorionic villi) from the placenta by aspiration through a transcervical catheter or transabdominal needle (7). These reproductive options may be used either by people clinically affected by MND, or individuals who had been identified as carrying an MND-linked gene variant by predictive (presymptomatic) testing. All plwMND in England are now eligible for whole genome sequencing, with reporting of variants identified in a panel of neurodegeneration linked genes (8). Genetic testing practices vary between countries, and there is no formal consensus among MND specialists. Research indicates that genetic testing is offered to individuals with a family history of MND, whereas the offer of testing to those with sporadic MND is inconsistent internationally (9, 10). With the advent of genetically targeted treatments for MND, there is increasing demand for whole genome sequencing, and more MND families will be found to have a monogenic cause which has reproductive implications.

In the English National Health Service (NHS), reproductive genetic counseling is a core task of clinical genetics services, and reproductive options such as PGT or PNT are managed by clinical genetics in conjunction with obstetrics or fertility clinics. Accessing these services requires non-specialist clinicians recognizing that their patient might be eligible for these, discussing the reproductive options with the family and offering a referral to clinical genetics. As such, awareness of reproductive options among non genetics professionals is vital to permit the identification of families which might benefit, and appropriate patterns of referral. However, there is evidence of limited knowledge and skills relating to reproductive medicine options amongst non-genetics healthcare professionals. For example, a survey of 201 US healthcare professionals involved in the care of families with genetic forms of breast or ovarian cancer found that only half were aware of PGT as an option (11). Klitzman et al. (12) found that 95% of the 163 neurologists that took part reported not feeling comfortable/qualified to discuss reproductive options with Huntington’s disease families. We could not identify any research investigating the knowledge and understanding of reproductive options of MND neurology clinicians.

Identifying barriers to reproductive genetic counseling was a key aspect of the survey. We therefore used the COM-B model for behavior change (13) as a framework to deductively map participant responses relating to barriers. The COM-B model specifies capability, opportunity, and motivation as key components capable of changing behavior, and modification of at least one of these components is required for any behavioral change to occur.

We undertook an online survey of neurology and genetics clinicians in the English NHS to assess their knowledge and skills relating to reproductive options for genetic forms of MND. We sought to understand current practice for reproductive counseling for families affected by genetic forms of MND, clinician knowledge of reproductive options, self-rated confidence in discussing these options, and perceived barriers to reproductive genetic counseling.

## Materials and methods

### Study design

A cross-sectional questionnaire survey, to assess clinician knowledge and skills in reproductive options, was delivered via *qualtrics,* between January 2024 and 1 May 2024. We followed the consensus-based checklist for reporting of survey studies. The survey was developed based on a review of the literature and a discussion with the research team (SA, AM, JH). Two consultant neurologists participated in a pilot, reviewed the survey and provided feedback on its clarity and comprehensiveness before it was distributed. The survey assessed knowledge on reproductive medicine techniques (6 questions) and confidence in discussing reproductive medicine options (6 questions). Furthermore, the survey questioned what barriers the participants perceived to discussing reproductive medicine options (1 question) and their preferred information resources to support discussions of reproductive medicine options (4 questions). The survey also collected demographic information and professional experience of the participants, and current reproductive counseling practices. MND and genetics clinicians were invited via email. Email invitations were distributed by the NIHR MND Clinical Studies Group, the ‘Predictive testing in Neurogenetic disease’ UK consortium, and MND Scotland. The reporting of the data is carried out according to the Consensus-Based Checklist for Reporting of Survey Studies (CROSS) (14) (Supplementary information 2).

### Data analyses

All statistical tests were performed using IBM SPSS Statistics (Version: 29.0.1.1(244)) and GraphPad. Descriptive statistics were used to summarize the frequency of survey responses and demographics. Likert scores were compared using the Mann-Whitney U test. A Chi squared test was used to identify differences in responses for clinical practice, knowledge, and training among clinician groups.

### Qualitative analysis of free text

Free text responses were deductively mapped onto COM-B constructs to explore any barriers to discussing reproductive medicine options. McGowan, Powell and French state that it isn’t useful to rigidly apply COM-B to a study, as this deductive approach will limit the analysis of results to those that fit into the COM-B framework (15). Instead, a flexible use of the COM-B model was used. The first author conducted this deductive mapping process according to the definition of each COM-B component.

## Results

### Participant characteristics

In total, 76 participants completed the survey including 45 neurology clinicians (17 consultant neurologists [22%], 8 neurology speciality registrars (StRs) [11%], 20 MND nurses [26%]), 31 clinical geneticists (15 clinical genetics consultants [20%] and 16 genetic counselors [21%]). MND care coordinators and MND specialist practitioners were included within the MND specialist nurse category. Participants were mostly female (64%, *n* = 49). Most reported having a special interest in MND (67%, *n* = 51) whilst only 17% (*n* = 13) of participants reported having a special interest in reproductive medicine. Participant characteristics are summarized in Table 1.

**Table 1.** Demographic details of participants.

Characteristics	Consultant neurologists (*n* = 17)	Clinical genetics consultants (*n* = 15)	Genetic counselors (*n* = 16)	MND specialist nurses (*n* = 20)	Neurology StR (*n* = 8)
Sex					
Male	12	5	1	2	6
Female	4	10	15	18	2
Prefer not to say	1	0	0	0	0
Age					
<30	0	0	1	0	2
30–39	1	0	5	5	5
40–49	6	12	8	7	1
50–59	7	1	1	6	0
60–69	3	2	1	2	0
70+	0	0	0	0	0
Years in substantive role					
<5	4	3	3	5	5
5–9	3	6	3	7	3
10–14	1	3	3	4	0
15–19	4	0	4	1	0
20–24	4	1	2	2	0
25+	1	2	1	1	0
Training in genetic counseling					
Yes	2	12	16	2	0
No	15	3	0	18	8
Training in reproductive medicine options for genetic conditions					
Yes	1	13	13	0	0
No	16	2	3	20	8
Special interest in MND					
Yes	12	9	7	20	3
No	5	6	9	0	5
Special interest in preimplantation genetic testing (PGT)/reproductive medicine					
Yes	0	0	9	4	0
No	17	15	7	16	8

### MND clinicians seldom discuss reproductive options with genetic MND families

**Figure 1.**
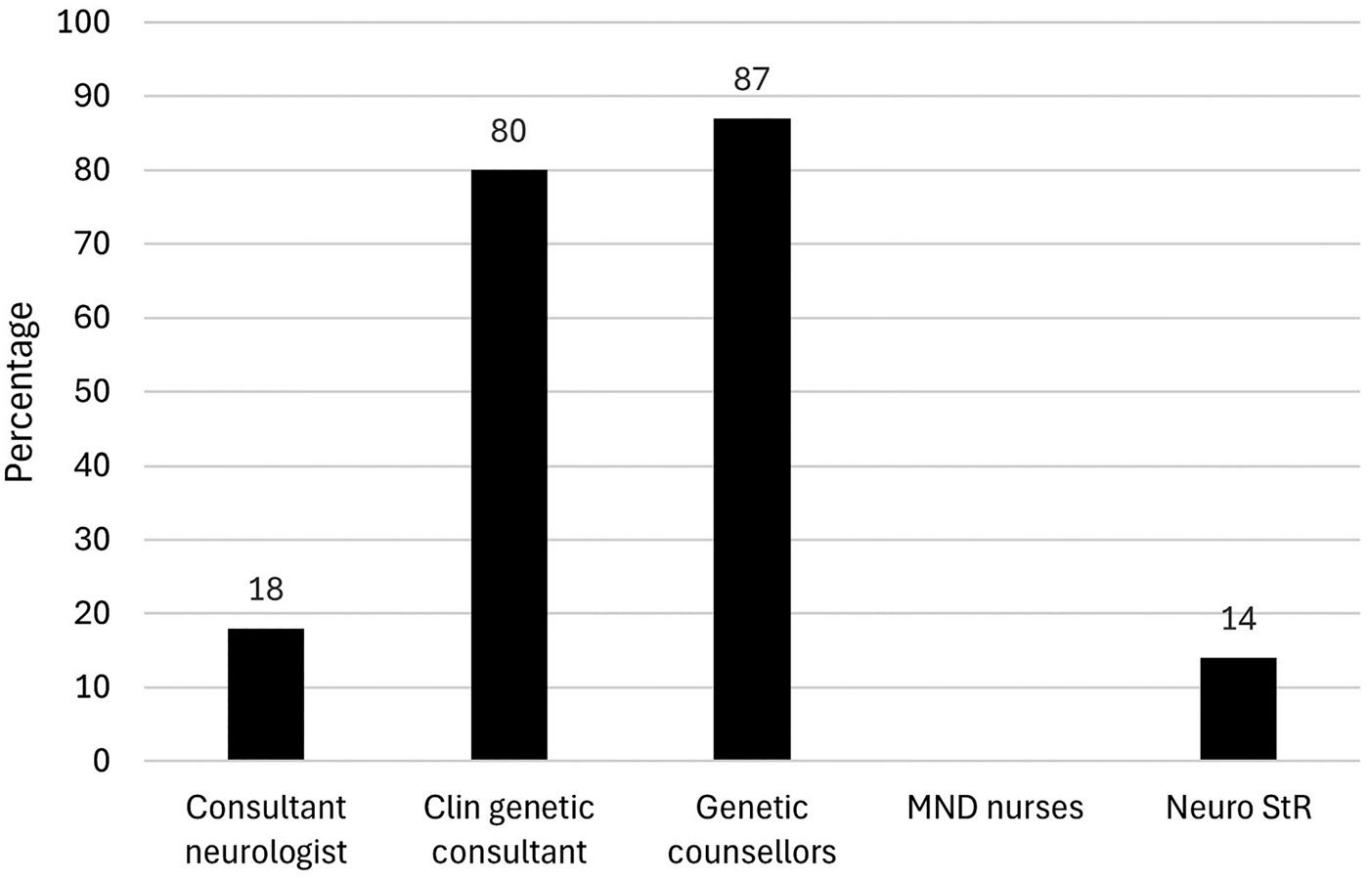
Bar chart displaying the percentage of each clinician group (neurology consultant, clinical genetics consultant, genetic counselor, MND nurse, neurology StR) reported to undertake reproductive genetic counseling with pwMND, a person carrying an MND gene variant or a person at risk of carrying an MND gene variant.

**Figure 2.**
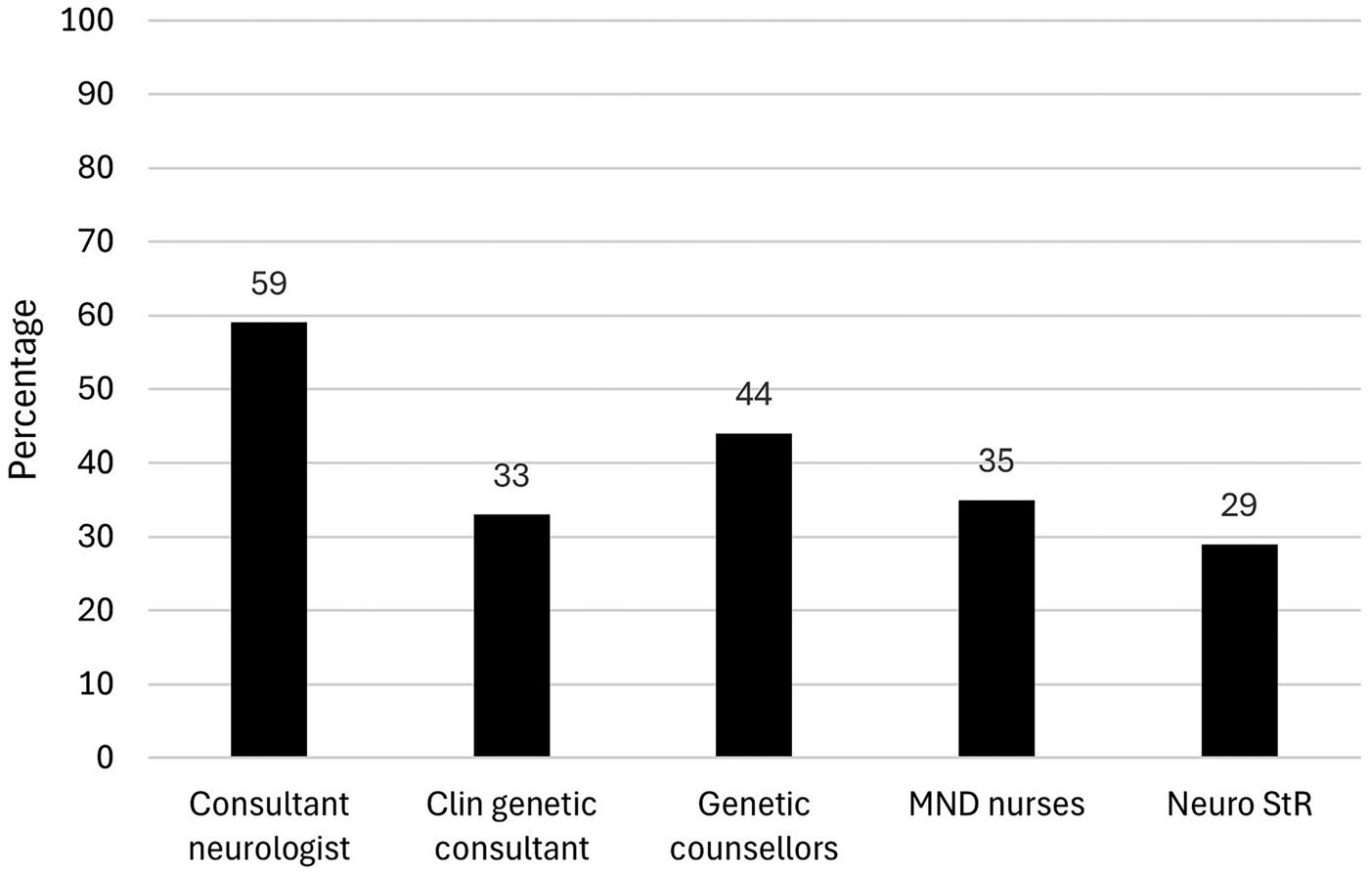
Bar chart displaying the percentage of each clinician group (neurology consultant, clinical genetics consultant, genetic counselor, MND nurse, neurology StR) reported to make a referral for reproductive genetic counseling for a person with pwMND, a person carrying an MND gene variant or a person at risk of carrying an MND gene variant.

Few neurology clinicians reported carrying out detailed reproductive genetic counseling for a person with an MND-linked gene variant. In total, 18% of consultant neurologists, 14% of neurology specialty registrars and no MND nurses had carried out reproductive genetic counseling. 59% of consultant neurologists had referred patients for reproductive genetic counseling, with the number dropping to 29% for neurology StRs and 35% for MND nurses (Table 2, Figures 1 and 2). Only 2 consultant neurologists had training in genetic counseling, and none had an interest in reproductive medicine.

**Table 2.** Clinical practice of reproductive genetic counseling and referrals.

	Number (%) who have undertaken detailed reproductive genetic counseling for:	Number (%) who have made a referral for reproductive genetic counseling for:
Consultant neurologist (n = 17)		
A person who has MND	2 (12%)	5 (29%)
A person who is considering predictive testing or has received a positive result for an MND-linked gene variant	3 (18%)	7 (41%)
A person who is at risk of carrying an MND-linked gene variant but has opted not to know their genetic status (e.g. non-disclosure/exclusion testing)	2 (12%)	6 (35%)
None of the above	14 (82%)	7 (41%)
Clinical genetics consultant (*n* = 15)		
A person who has MND	6 (40%)	2 (13%)
A person who is considering predictive testing or has received a positive result for an MND-linked gene variant	10 (67%)	3 (20%)
A person who is at risk of carrying an MND-linked gene variant but has opted not to know their genetic status (e.g. non-disclosure/exclusion testing)	2 (13%)	1 (7%)
None of the above	3 (20%)	10 (67%)
Genetic counselor (*n* = 16)		
A person who has MND	4 (25%)	0
A person who is considering predictive testing or has received a positive result for an MND-linked gene variant	14 (88%)	6 (38%)
A person who is at risk of carrying an MND-linked gene variant but has opted not to know their genetic status (e.g. non-disclosure/exclusion testing)	9 (56%)	4 (25%)
None of the above	2 (13%)	9 (56%)
MND specialist nurse (*n* = 20)		
A person who has MND	0	4 (20%)
A person who is considering predictive testing or has received a positive result for an MND-linked gene variant	0	3 (15%)
A person who is at risk of carrying an MND-linked gene variant but has opted not to know their genetic status (e.g. non-disclosure/exclusion testing)	0	2 (10%)
None of the above	20 (100%)	13 (65%)
Neurology StR (*n* = 7)		
A person who has MND	1 (14%)	2 (29%)
A person who is considering predictive testing or has received a positive result for an MND-linked gene variant	0	1 (14%)
A person who is at risk of carrying an MND-linked gene variant but has opted not to know their genetic status (e.g. non-disclosure/exclusion testing)	0	0
None of the above	6 (86%)	5 (71%)

Free text responses demonstrated that barriers to discussing reproductive medicine options were closely associated with four (out of six) COM-B sub-components: physical capability, psychological capability, physical opportunity, and reflective motivation. The most commonly reported barrier was lack of knowledge/training (psychological capability construct). Another commonly reported barrier was the lack of a clear pathway and uncertainty around whose responsibility it was to have these discussions, with one participant stating they have a “vague idea that clinical genetics will do it” (reflective motivation construct). The participant quotes mapped onto the COM-B model are presented in Table 3.

**Table 3.** Participant quotations related to barriers in discussing reproductive options and training needs mapped into the COM-B model.

COM-B (sub)component	Free text quotations
Consultant neurologists	
Psychological capability	“Lack of knowledge on clinicians part” “lots of anxiety, knowledge of what’s out there is lacking”
Physical opportunity	“Waiting time for clinical genetics appointment” “Time in the clinic appointment can be pressured i.e. there is not enough time to facilitate the discussion” “time to discuss with large numbers of people” “Barriers no worse than other referrals to specialised services; principally the waits involved”
Reflective motivation	“Local service provision variance” “unsure about funding.fact that these are not ‘patients’ so are taking up space in patient clinic” “none, but our population is elderly so no opportunities to do so” “Not really though I suspect that this would be something entirely under the remit of the neuromuscular specialist clinic rather than general neurologists”
Clinical genetic consultants	
Psychological capability	“Only the knowledge of the ‘counsellor’ – but in general this workload is only handled by experienced clinicians.” “Yes; education. Non-genetics specialists don’t routinely discuss pregnancy related issues and, unless there is a specific reason to do so, would not include this in their practice” “Main barrier is not having people referred to us so we can have those discussions (i.e. relatives of those who have a +’ve gene test).”
Physical opportunity	“Main barrier is not having people referred to us so we can have those discussions (i.e. relatives of those who have a +’ve gene test).” “Long waiting times for genetic appointments”
Reflective motivation	“Lack of confidence amongst neurology colleagues to discuss intricacies of PGT etc” “Non-genetics specialists don’t routinely discuss pregnancy related issues and, unless there is a specific reason to do so, would not include this in their practice”
Genetic counselors	
Physical capability	“I am concerned more than not all MND patients are offered genetic testing by neurologists when they could be offered this, and I suspect that prenatal options are rarely brought up, unless by the patient themselves”
Psychological capability	“Only lack of knowledge or how to signpost to clinical genetics” “In our region we now encourage diagnostic testing to take place outside of clinical genetics, and for affected patients to be referred to us if they receive a positive result from this testing. However we still receive a number or referrals for unaffected relatives of affected individuals where the affected person has not had a gene test themselves. I therefore think that limited understanding / resource availability to carry out diagnostic testing in mainstream services can sometimes be a barrier for younger people who are considering their reproductive options” “If clinicians testing patients are not fully educated about reproductive options, they may not inform their patients about them” “or their understanding of the meaning of a predictive test, or exclusion tests”
Physical opportunity	“Lack of time in appointments” “In our region we now encourage diagnostic testing to take place outside of clinical genetics, and for affected patients to be referred to us if they receive a positive result from this testing. However we still receive a number or referrals for unaffected relatives of affected individuals where the affected person has not had a gene test themselves. I therefore think that limited understanding/resource availability to carry out diagnostic testing in mainstream services can sometimes be a barrier for younger people who are considering their reproductive options”
Reflective motivation	“Short life expectancy and declining health of someone with MND” “I am concerned more than not all MND patients are offered genetic testing by neurologists when they could be offered this, and I suspect that prenatal options are rarely brought up, unless by the patient themselves” “and the focus is often on the ‘now’ so if they are not actively family planning it often falls to the bottom of the list when there are so many other issues to discuss” “Barriers to access may include i) referrers uncertainty about the availability of prenatal/PGT for later onset conditions”
MND specialist nurses	
Physical capability	“Many. This is something that I do not have any experience in and would feel unable to support my patients with the appropriate knowledge and skill in delivery”
Psychological capability	“I am not trained to do so, this should be done by a genetics service” “Just need some formalized training for team members who are not nurses” “MND & genetic in same conversation / I would require support to have up to date knowledge” “Staff knowledge, experience and training” “time, knowledge, availability of services, funding etc” “Lack of awareness from professionals.” “in our neurology service, it it knowledge, expertise and time” “Lack of knowledge in this subject area” “Lack of education on the issue, I would like to know more on the subject, what pathways exist in services that are ‘doing it well’ and how we could transfer ideas to develop our own pathway.” “lack of knowledge”
Physical opportunity	“We no longer have a lead Consultant and our patients are being seen by general Neurologists. I feel this is a barrier” “distance to access services” “staff time” “time, knowledge, availability of services, funding etc” “Availability of specialist practitioners to fully discuss and inform patients/relatives. The slow process of genetic testing.” “The backlog in seeing patients by the clinical genetics team.” “in our neurology service, it it knowledge, expertise and time” “I wonder about resources, if this is a new aspect of our service will this require additional capacity to both staff and lab time”
Neurology StRs	
Physical capability	“Generally lack of knowledge and skills on my part, although possible for some genetic conditions, the genetics of some MND genes seems difficult for a non-specialist to give accurate information for”
Psychological capability	“Generally lack of knowledge and skills on my part, although possible for some genetic conditions, the genetics of some MND genes seems difficult for a non-specialist to give accurate information for” “lack of knowledge of how the referral pathway and service works” “lack of adequate training” “Lack of knowledge”
Reflective motivation	“Lack of clear pathway, uncertain whose responsibility. Vague idea that ‘clinical genetics will do it’”

### MND clinicians have low self-reported knowledge and confidence in discussing reproductive options

**Figure 3.**
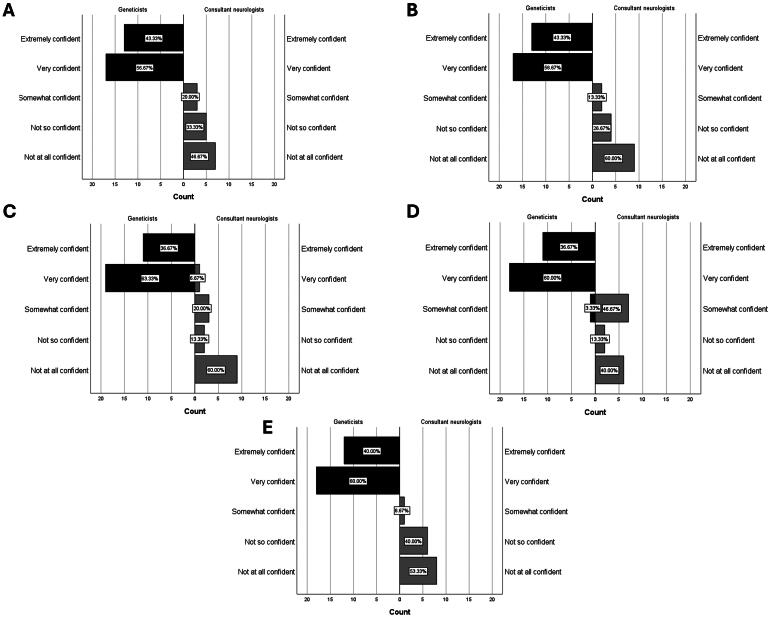
Self-reported confidence in explaining reproductive medicine options. Pyramid blots illustrate geneticists (black) and consultant neurologists (grey) responses on a 5-point Likert scale. (A) Explaining the process of amniocentesis (B) Explaining the process of chorionic villus sampling (C) Explaining the process of noninvasive prenatal testing (D) Explaining the process of preimplantation genetic testing (D) Explaining the risks and benefits of various reproductive options.

Geneticists demonstrated having greater knowledge of reproductive medicine options and the eligibility around accessing them than MND clinicians. A chi-squared test demonstrated that consultant neurologists, MND specialist nurses and neurology StRs scored significantly lower in each clinical scenario question than geneticists (Table 4). Further, all clinical genetic consultants and nearly all genetic counselors rated themselves as either “extremely confident” or “very confident” in explaining each reproductive medicine option to patients. Only a very small minority of MND clinicians rated themselves as “extremely confident” or “very confident” in explaining each reproductive medicine option. A Mann–Whitney *U* test demonstrated that MND clinicians rated themselves significantly less confident in explaining each reproductive medicine option item than geneticists (Figure 3 and Table 5).

**Table 4.** Clinician responses to clinical scenarios.

	Number (%) of participants who answered “I don’t know” or gave an incorrect answer				
Questions	Consultant Neurologist (*n* = 17)	Clinical genetics consultant (*n* = 15)	Genetic counselors (*n* = 16)	MND nurses (*n* = 20)	Neurology StR (*n* = 7)
Can a man with MND and an autosomal dominant family history, but no identified causal gene variant, access PGT/PNT?	6** (35%)	0	0	16*** (80%)	6** (86%)
Can a woman with a SOD1 causal variant request termination of a fetus found to carry the SOD1 variant through prenatal testing?	6* (35%)	1 (7%)	0	14*** (70%)	4** (57%)
Can a couple without children, in which the man has MND and a causal SOD1 variant access NHS funded PGT?	8** (47%)	2 (13%)	0	15*** (75%)	5** (71%)
Will a woman with a SOD1 causal variant who has an amniocentesis have an increased risk of miscarriage?	6** (35%)	0	0	13*** (65%)	2* (29%)
Can a presymptomatic man with a variant of uncertain significance in SOD1 use PGT?	13** (76%)	2 (13%)	0	19*** (95%)	4** (57%)
Can a woman with MND and a c9orf72 expansion whose husband has a healthy child from another marriage access NHS funded PGT?	11** (65%)	3 (20%)	0	17*** (85%)	6** (86%)

* *p* < 0.005 on Chi Squared test. Geneticists compared to consultant neurologists or neurology StRs.

** *p* < 0.001 on Chi Squared test. Geneticists compared to consultant neurologists or neurology StRs.

*** *p* < 0.0001 on Chi Squared test. Geneticists compared to MND specialist nurses.

**Table 5.** The number of individuals in each clinician group who reported confidence for each reproductive option item and overall median confidence score for each clinician group for each reproductive medicine option.

Likert scale statements	Reported “extremely confident” or “very confident”				
	Consultant Neurologist (*n* = 17)	Clinical genetics consultant (*n* = 15)	Genetic counselors (*n* = 16)	MND nurses (*n* = 20)	Neurology StR (*n* = 7)
I feel confident explaining the process of amniocentesis to a patient.	0	15 (100%)	16 (100%)	1 (6%)	0
I feel confident explaining the process of chorionic villus sampling to a patient.	0	15 (100%)	16 (100%)	1 (6%)	0
I feel confident explaining the process of noninvasive prenatal testing to a patient.	1 (7%)	15 (100%)	16 (100%)	0	0
I feel confident explaining the process of preimplantation genetic testing to a patient.	0	15 (100%)	15 (94%)	0	0
I feel confident explaining the risks and benefits of various reproductive medicine options.	0	15 (100%)	16 (100%)	0	0

**Table:** 

Likert scale statements	Medium scores for each clinician group (1 = not at all confident, 5 = extremely confident)			
	Consultant Neurologist (*n* = 17)	Geneticists (genetics consultant and genetic counselors) (*n* = 31)	MND nurses (*n* = 20)	Neurology StR (*n* = 7)
I feel confident explaining the process of amniocentesis to a patient.	2*	4	1**	2*
I feel confident explaining the process of chorionic villus sampling to a patient.	1*	4	1**	1*
I feel confident explaining the process of noninvasive prenatal testing to a patient.	1*	4	1**	1*
I feel confident explaining the process of preimplantation genetic testing to a patient.	2*	4	1**	2*
I feel confident explaining the risks and benefits of various reproductive medicine options.	1*	4	1**	1*

* *p* < 0.001 on Mann–Whitney *U* test. Geneticists compared to consultant neurologists or neurology StRs.

** *p* < 0.001 on Mann–Whitney *U* test. Geneticists compared to MND specialist nurses.

Free text responses were used to seek further information on confidence levels and where additional training would be beneficial for discussing reproductive options. Responses were deductively mapped onto COM-B constructs. When asked for further information on their confidence levels and to see where additional training would be beneficial for discussing reproductive medicine options, participant responses were closely associated with three COM-B sub-components: psychological capability, physical opportunity, and reflective motivation. Responses from MND nurses and neurology StRs were associated with capability (psychological) where their confidence stemmed from a lack of knowledge or training. For example, one MND nurse stated, “I would feel very overwhelmed discussing this in any detail. Therefore, I feel I would need full training from novice level”. Responses from consultant neurologists were associated with motivation (reflective), with some suggesting it was not part of their role to explain reproductive medicine options. One consultant neurologist expressed, “this is not my role so I don’t really need additional training”. The participant quotes mapped onto the COM-B model are presented in Table 6.

**Table 6.** Participant quotations related to confidence in discussing reproductive options and training needs mapped into the COM-B model.

COM-B (sub)component	Free text quotations
Consultant neurologists	
Physical opportunity	“I would not have capacity to talk about this”
Reflective motivation	“I understand the basic concepts, but as a neurologist I would always defer to colleagues in clinical genetics for these conversations” “Do not think the fine detail of risk/benefit of reproductive medicine can realistically be a core accredited part of neurologists’ curriculum, just as, for example, geneticists would not be routinely trained to advise parents on the risk/benefit of orthopedic options for children born with skeletal problems.” “this is not my role so I don’t really need additional training” “I am a neurologist, unsure why the above questions being asked of me” “I do not need more training as this is not something I do and we have access to a genetics team.” “I don’t think most people will do MND genetics before they refer to the neuromuscular clinic and so unless MND specialists most will never know of any genetic result”
Genetic counselors	
Reflective motivation	“The advice changes over time so I would not describe myself as extremely confident”
MND specialist nurses	
Psychological capability	“I am a HD nurse also, but only in both posts for just over a year so although I know there are options until presented with a scenario and need to learn in depth for case specific scenarios as they are very complex.” “I would not discuss without training, the answers previously on the questionnaire are my thoughts i would NEVER advise on the scenarios in real life” “This has not been within my specialist role, therefore I would feel very overwhelmed discussing this in any detail. Therefore, I feel I would need full training from novice level.” “It’s a new area for us therefore a package of additional training and support would be v welcome” “I am not confident because I do not have enough knowledge in these areas to explain”
Neurology StRs	
Psychological capability	“Not covered since medical school” “I think from a neurologist’s perspective we probably need some straightforward training on what we can offer PGT for, common genes that can’t be tested for, and the general process to inform the patient of what they can expect at the point of referral” “lack of training”

### MND clinicians lack information resources to support reproductive option discussions

Participants were asked to rate on a likert scale to what extent they agreed/disagreed with the following statement “I have access to adequate information resources to support discussions on reproductive medicine options with MND families”. Only a minority of MND clinicians (43% neurology consultants, 14% neurology StRs and 19% MND nurses) felt they had sufficient resources to support these discussions. 61% of clinical genetic consultants and 80% of genetic counselors believed they had access to adequate information resources (Table 7).

**Table 7.** Free text responses on information resources.

What resources do you currently have access to?	
Consultant neurologists	“None, except a good working relationship with genetics colleagues” “Not sure, not required yet.” “Self sourced. Usually through the genetics service. “no idea and tbh not time to find out” “None” “I do not need these”
Neurology StRs	“I am not sure I have access to any” “none that I’m aware of” “Asking senior colleagues” “None”
MND specialist nurses	“A patient asked me about this last week (first time it has come up) I frantically googled to find out even if pre implantation manipulation is available on NHS” “NONE” “None” “None” “None” “None” “We don’t have any resources.”
Clinical genetic consultants	“Genetic Counselors!” “genetic counselors!” “Specialist genetic counseling team” “None - just my own letters”
Genetic counselors	• [No free text responses]
Please elaborate on what information resources would be helpful	
Consultant neurologists	“No experience of any of the above available in my Trust at the point of care for MND” “it has to be something I don’t have involvement with.” “No idea, I don’t think neurologists have role in this”
Neurology StRs	• “Also focused training for clinicians would give them confidence in discussing this in clinic”
MND specialist nurses	“Staff Training” “Any resources frankly but staff training particularly” “Additional training for nurses to support discussions and directions to good online resources” “Training resources aimed at professionals and patients separately” “I would like to expand my knowledge so any resources would be helpful”
Clinical genetic consultants	• “Training videos for clinicians”
Genetic counselors	“training resources for clinicians outside genetics to be able to explain who is eligible for predictive testing and prenatal testing” “patient decision aid and more training for people in neurology would be helpful”

Participants were asked to rate the following information resources (information leaflets; web-based information; patient information videos; a patient decision aid and training resources) from 1 (the preferred option) to 5 (the least preferred option). Among MND clinicians, information leaflets were the top preference, with 14 clinicians rating them as the most preferred option. Training resources were the second most favored, with 8 clinicians choosing them as their top option. Geneticists also preferred information leaflets the most, with 10 rating them as their preferred choice. However, for geneticists, web-based information was the second most preferred option, with 7 selecting it as their top choice, while training resources ranked third.

Free text comments from all participant groups also reinforced the need for training resources around reproductive options. One MND specialist nurse emphasized this by stating, “A patient asked me about this last week, I frantically googled to find out even if pre implantation manipulation is available on the NHS”. Other participants echoed similar sentiments, expressing the desire for training resources. For instance, when asked what resources would be helpful, a neurology StR expressed, “focused training for clinicians to give them confidence in discussing this in the clinic”. Furthermore, all clinician groups reported lacking information resources, and no respondents mentioned having any physical materials available.

## Discussion

We found that few MND clinicians reported undertaking reproductive genetic counseling with either plwMND or asymptomatic gene variant carriers. Less than half of MND clinicians stated they had referred a patient for reproductive genetic counseling. In addition, MND clinicians report low levels of knowledge of reproductive options, low self-reported confidence in reproductive counseling and inadequate information resources to support clinic discussions. Lack of knowledge, lack of awareness of the different options, lack of clinic time and uncertainty around issues such as funding for PGT were also described, highlighting that neurology clinicians face multiple, overlapping barriers to offering reproductive counseling or referral.

Our findings are in keeping with the literature on reproductive genetic counseling for other conditions.

A survey of North American internal medicine physicians found that 4.9% had discussed PGT with patients, and only 7% felt qualified to answer questions on the technique (16). For many common genetic conditions (e.g cystic fibrosis, genetic breast cancer) surveyed internal medicine clinicians were unsure whether or not to offer referral for PGT (17). A survey of oncology professionals in the Netherlands identified that fewer than half had referred eligible patients for PGT (18). There is evidence that low referral rates for reproductive genetic counseling are associated with lower rates of knowledge and training in reproductive options. In addition, our findings of low confidence in discussing reproductive options is consistent with a recent survey of neurology clinicians where a lack of confidence in MND genomic testing and low reported genomic medicine skills were reported. Similarly, multiple barriers were identified such as lack of time to discuss genomic testing in clinics and a lack of training (19).

We found that only a minority of neurology clinicians had referred a plwMND or an asymptomatic individual with a positive predictive test result for reproductive genetic counseling. Whilst this may be in part due to a lack of need in their patients, this was associated with low knowledge scores on reproductive options for MND families. None of the neurology consultants or StRs felt confident to explain amniocentesis, chorionic villus sampling or PGT to patients. One MND specialist nurse felt confident to explain these options. None of the neurology clinicians felt confident to explain the pros and cons of each option. It seems reasonable to suggest that low levels of knowledge and confidence relating to discussing reproductive options may act as a barrier to clinicians discussing reproductive options with MND families or offering referral to clinical genetics. Indeed, this was explicitly identified as a barrier in free-text responses.

Free text responses identified several key barriers to discussing reproductive options. Factors related to psychological capability, physical opportunity and reflective motivation were the most commonly cited barriers, including specifically a lack of clinician knowledge (psychological capability), inadequate time in the appointment, a lack of resources (physical opportunity), limited confidence and role ambiguity (reflective motivation). These barriers were consistent with wider survey findings, with all MND clinicians scoring significantly lower in each knowledge question than geneticists. Indeed, only a small minority of MND clinicians rated themselves as “extremely confident” or “very confident” in explaining each reproductive medicine option. Further, the majority of MND clinicians (57% neurology consultants, 86% neurology StRs and 81% MND nurses) felt that they did not have enough resources to support discussions on reproductive medicine options with MND families – a finding which builds on recent research suggesting a dearth of resources to support genetic testing in MND generally (19). Many of these barriers may be addressed with appropriate training and resources.

In the English NHS, neurology clinicians from MND specialist clinics report low knowledge and confidence in reproductive medicine options for families affected by genetic forms of MND, a limited use of referral to specialist genetics services. Our paper has implications for clinical practice and service development both nationally and internationally. Services must make sure that clinicians have appropriate training and resources to support discussions of reproductive options and appropriate referrals to clinical genetics. They may be ideally suited to this latter role given they play a key role in patient care and are able to build rapport over time. Training curricula should be revised to include relevant topics such as MND genetics and the availability of reproductive options. The additional complexities of such options in MND, given the varying pentrance of MND gene variants, warrants the development of disease-specific materials for clinicians and patients. Additionally, the varying knowledge and confidence of professionals regarding reproductive medicine options in MND highlights the need for clear guidelines for them to refer to. The lack of resources to support reproductive option discussions suggests that patients may not receive the essential information and guidance needed to make informed decisions about their reproductive options. Developing resources such as videos or information leaflets could make patients aware of these options and address this gap. While MND clinicians may not be specialists in reproductive genetic counseling, they should be aware of these reproductive options and refer their patients to clinical genetics so that families can benefit.

## Limitations

The knowledge of reproductive options was assessed using an instrument that had not undergone validation. Nevertheless, as far as we know, there is currently no validated tool available for measuring the knowledge of this. Another limitation is the small sample size as this may weaken the external validity and affect the generalizability of the results.

## Supplementary Material

Supplementary_data.docx
