# Supplementary material for: Neurologists’ understanding of reproductive medicine options for genetic forms of motor neuron disease
Source: Amyotroph Lateral Scler Frontotemporal Degener. 2024 Oct 18;26(3-4):331–42. doi: 10.1080/21678421.2024.2416677 (PMC12011025; doi:10.1080/21678421.2024.2416677)
Supplement: Supplementary_data.docx [file IAFD_A_2416677_SM6389.docx]

**Supplementary information.**

**Supplementary information 1: Survey**

Clinician survey final - reproductive medicine

This survey is about your experiences of and knowledge around reproductive medicine options in Motor Neuron Disease (MND), with a focus on prenatal testing (PNT) and preimplantation genetic testing (PGT). We will use your responses to inform the development of co-produced information resources for patients and training packages for MND clinicians to support access to reproductive medicine options.

First, you will be asked for some information about you and your role. Next, you will be asked about your current MND reproductive counselling practices. The third section is about barriers you feel impede access to these options in MND. The fourth section focuses on your knowledge around techniques such as PNT and PGT. The fifth section focuses on confidence in discussing reproductive options. The final section is about information and support.

You will be asked to fill in a consent form in the next section before you begin.

Please read the participant information sheet before signing the consent form (presented in Qualtrics).

You will be offered the chance to be entered into a prize draw to win a £50 Amazon voucher to thank you for your time.

The survey will take approximately 5-10 minutes to complete.

If you would like to be entered into the prize draw for a £50 voucher, please leave your email address below.

CONSENT FORM (presented in Qualtrics)

DEMOGRAPHICS

In this section you will be asked about you and your role.

What is your age?

< 30

30 - 39

40 - 49

50 - 59

60 - 69

70 +

What is your sex?

Male

Female

Other

Prefer not to say (please specify)

What is your specialism?

Consultant neurologist

Neurology StR

Clinical genetics consultant

Clinical genetics StR

Genetic counsellor

MND Nurse

Other (please specify)

Years in substantive role

< 5

5 - 9

10 - 14

15 - 19

20 - 24

25 +

Do you have any training in genetic counselling (e.g. CCT, MSc, CPD course)?

Yes

No

Do you have any training in reproductive medicine options for genetic conditions?

Yes

No

Do you have a special interest in MND?

Yes

No

Do you have a special interest in preimplantation genetic diagnosis (PGD) / reproductive medicine?

Yes

No

CLINICAL PRACTICE

In this section you will be asked about your current MND reproductive counselling practices.

In your service, who discusses reproductive medicine options with MND families? (TICK ALL THAT APPLY)

Consultant neurologist

Consultant clinical genetics

Genetic counsellor

MND specialist nurse

Neurology StR

Clinical genetics StR

Other (please specify)

Have you undertaken detailed reproductive genetic counselling (discussed options such as PGT or PNT) for the following individuals (TICK ALL THAT APPLY):

A person that has MND.

A person who is considering predictive testing or has received a positive result for an MND-linked gene variant.

A person who is at risk of carrying an MND-linked gene variant but has opted not to know their genetic status (e.g. non-disclosure/exclusion testing).

None of the above.

Have you made a referral for reproductive genetic counselling for the following individuals (TICK ALL THAT APPLY):

A person that has MND.

A person who is considering predictive testing or has received a positive result for an MND-linked gene variant.

A person who is at risk of carrying an MND-linked gene variant but has opted not to know their genetic status (e.g. non-disclosure/exclusion testing).

None of the above.

Do you have an established local pathway for referring people for reproductive genetic counselling (e.g to clinical genetics)?

Yes

No

I don’t know

BARRIERS

In this section you will be asked about barriers you feel impede access to these options in MND.

Do you perceive any barriers to discussing reproductive medicine options?

Yes

No

If yes, please provide details.

_________________________________________________________________________________________________________________________________________________________________________________________________________________________________________________________________________________________________________________________________________________________

CLINICAL SCENARIOS TO TEST KNOWLEDGE

In this section you will be given clinical scenarios to test your knowledge around techniques such as PNT and PGT.

Can a man with MND and an autosomal dominant family history, but no identified causal gene variant, access reproductive medicine options?

Yes

No

I don’t know

Can a woman with a SOD1 causal variant request termination of a foetus found to carry the SOD1 variant through PNT?

Yes

No

I don’t know

Can a couple without children, in which the man has MND and a causal SOD1 variant access NHS funded PGD?

Yes

No

I don’t know

Will a woman with a SOD1 causal variant who has an amniocentesis have an increased risk of miscarriage?

Yes

No

I don’t know

Can an asymptomatic man with a variant of uncertain significance in SOD1 use PGD?

Yes

No

I don’t know

Can a woman with MND and a c9orf72 expansion whose husband has a healthy child from another marriage access NHS funded PGD?

Yes

No

I don’t know

SKILLS/CONFIDENCE

In this section you will be asked about your confidence in discussing reproductive medicine options.

How confident do you feel explaining reproductive medicine options?

(1 = not at all confident, 2 = not so confident, 3 = somewhat confident, 4 = very confident, 5 = extremely confident)

I feel confident explaining the process of amniocentesis to a patient. 1-5.

I feel confident explaining the process of chorionic villus sampling to a patient. 1-5.

I feel confident explaining the process of preimplantation testing to a patient. 1-5.

I feel confident explaining the process of non-invasive prenatal testing to a patient. 1-5.

I feel confident in explaining the risks and benefits of various reproductive medicine options. 1-5

Please provide any further details (e.g. on aspects you are confident/not confident in and why, or areas where additional training would be beneficial)?

_________________________________________________________________________________________________________________________________________________________________________________________________________________________________________________________________________________________________________________________________________________________

INFORMATION RESOURCES ON RDM

In this section you will be asked about your opinion on information resources on reproductive decision making.

Access to information resources
(1 = strongly disagree, 2 = disagree, 3 = neither agree or disagree, 4 = agree, 5= strongly agree)

I have access to adequate information resources (e.g. leaflets) to support discussions on reproductive medicine options with MND families. 1 – 5.

What information resources do you currently have access to around reproductive medicine options? (please specify)

_________________________________________________________________________________________________________________________________________________________________________________________________________________________________________________________________________________________________________________________________________________________

Please rank these information resources to support discussions of reproductive medicine options in order of preference:

Information leaflets

Web based information i.e. information sheets/guides

Patient information videos

A patient decision aid

Training resources

Please elaborate on what information resources would be helpful.

_________________________________________________________________________________________________________________________________________________________________________________________________________________________________________________________________________________________________________________________________________________________

We thank you for your time spent taking this survey.

Your response has been recorded.

END OF SURVEY

**Supplementary information 2: CROSS guideline**

**Checklist for Reporting Of Survey Studies (CROSS)**

| **Section/topic** | **Item** | **Item description** | **Reported on page #** |
| --- | --- | --- | --- |
| **Title and abstract** | | |  |
| Title and abstract | 1a | State the word “survey” along with a commonly used term in title or abstract to introduce the study’s design. | 2 |
|  | 1b | Provide an informative summary in the abstract, covering background, objectives, methods, findings/results, interpretation/discussion, and conclusions. | 2 |
| **Introduction** | | |  |
| Background | 2 | Provide a background about the rationale of study, what has been previously done, and why this survey is needed. | 3 |
| Purpose/aim | 3 | Identify specific purposes, aims, goals, or objectives of the study. | 3 |
| **Methods** | | |  |
| Study design | 4 | Specify the study design in the methods section with a commonly used term (e.g., cross-sectional or longitudinal). | 4 |
|  | 5a | Describe the questionnaire (e.g., number of sections, number of questions, number and names of instruments used). | 4 |
| Data collection methods | 5b | Describe all questionnaire instruments that were used in the survey to measure particular concepts. Report target population, reported validity and reliability information, scoring/classification procedure, and reference links (if any). |  |
|  | 5c | Provide information on pretesting of the questionnaire, if performed (in the article or in an online supplement). Report the method of pretesting, number of times questionnaire was pre-tested, number and demographics of participants used for pretesting, and the level of similarity of demographics between pre-testing participants and sample population. | 4 |
|  | 5d | Questionnaire if possible, should be fully provided (in the article, or as appendices or as an online supplement). | 22 |
| Sample characteristics | 6a | Describe the study population (i.e., background, locations, eligibility criteria for participant inclusion in survey, exclusion criteria). |  |
|  | 6b | Describe the sampling techniques used (e.g., single stage or multistage sampling, simple random sampling, stratified sampling, cluster sampling, convenience sampling). Specify the locations of sample participants whenever clustered sampling was applied. | 4 |
|  | 6c | Provide information on sample size, along with details of sample size calculation. |  |
|  | 6d | Describe how representative the sample is of the study population (or target population if possible), particularly for population-based surveys. |  |
| Survey  administration | 7a | Provide information on modes of questionnaire administration, including the type and number of contacts, the location where the survey was conducted (e.g., outpatient room or by use of online tools, such as SurveyMonkey). | 4 |
|  | 7b | Provide information of survey’s time frame, such as periods of recruitment, exposure, and follow-up days. | 4 |
|  | 7c | Provide information on the entry process:  –>For non-web-based surveys, provide approaches to minimize human error in data entry.  –>For web-based surveys, provide approaches to prevent “multiple participation” of participants. |  |
| Study preparation | 8 | Describe any preparation process before conducting the survey (e.g., interviewers’ training process, advertising the survey). |  |
| Ethical considerations | 9a | Provide information on ethical approval for the survey if obtained, including informed consent, institutional review board [IRB] approval, Helsinki declaration, and good clinical practice [GCP] declaration (as appropriate). |  |
|  | 9b | Provide information about survey anonymity and confidentiality and describe what mechanisms were used to protect unauthorized access. |  |
| Statistical  analysis | 10a | Describe statistical methods and analytical approach. Report the statistical software that was used for data analysis. | 4 |
|  | 10b | Report any modification of variables used in the analysis, along with reference (if available). |  |
|  | 10c | Report details about how missing data was handled. Include rate of missing items, missing data mechanism (i.e., missing completely at random [MCAR], missing at random [MAR] or missing not at random [MNAR]) and methods used to deal with missing data (e.g., multiple imputation). |  |
|  | 10d | State how non-response error was addressed. |  |
|  | 10e | For longitudinal surveys, state how loss to follow-up was addressed. | N/A |
|  | 10f | Indicate whether any methods such as weighting of items or propensity scores have been used to adjust for non-representativeness of the sample. |  |
|  | 10g | Describe any sensitivity analysis conducted. |  |
| **Results** | | |  |
| Respondent characteristics | 11a | Report numbers of individuals at each stage of the study. Consider using a flow diagram, if possible. |  |
|  | 11b | Provide reasons for non-participation at each stage, if possible. |  |
|  | 11c | Report response rate, present the definition of response rate or the formula used to calculate response rate. |  |
|  | 11d | Provide information to define how unique visitors are determined. Report number of unique visitors along with relevant proportions (e.g., view proportion, participation proportion, completion proportion). |  |
| Descriptive  results | 12 | Provide characteristics of study participants, as well as information on potential confounders and assessed outcomes. | 6/9 |
| Main findings | 13a | Give unadjusted estimates and, if applicable, confounder-adjusted estimates along with 95% confidence intervals and p-values. |  |
|  | 13b | For multivariable analysis, provide information on the model building process, model fit statistics, and model assumptions (as appropriate). |  |
|  | 13c | Provide details about any sensitivity analysis performed. If there are considerable amount of missing data, report sensitivity analyses comparing the results of complete cases with that of the imputed dataset (if possible). |  |
| **Discussion** | | |  |
| Limitations | 14 | Discuss the limitations of the study, considering sources of potential biases and imprecisions, such as non-representativeness of sample, study design, important uncontrolled confounders. | 7 |
| Interpretations | 15 | Give a cautious overall interpretation of results, based on potential biases and imprecisions and suggest areas for future research. | 6 |
| Generalizability | 16 | Discuss the external validity of the results. | 7 |
| **Other sections** | | |  |
| Role of funding source | 17 | State whether any funding organization has had any roles in the survey’s design, implementation, and analysis. | 8 |
| Conflict of interest | 18 | Declare any potential conflict of interest. | 8 |
| Acknowledgements | 19 | Provide names of organizations/persons that are acknowledged along with their contribution to the research. | 8 |
